# Does climate change transform military medicine and defense medical support?

**DOI:** 10.3389/fpubh.2023.1099031

**Published:** 2023-05-04

**Authors:** Yohan Robinson, Amir Khorram-Manesh, Niclas Arvidsson, Cave Sinai, Fabian Taube

**Affiliations:** ^1^Centre for Disaster Medicine, Gothenburg University, Gothenburg, Sweden; ^2^Joint Centre for Defence Medicine, Swedish Armed Forces, Gothenburg, Sweden

**Keywords:** military medicine, defense medical support, climate change, medical logistics, preventive medicine

## Abstract

**Background:**

Climate change has effects on multiple aspects of human life, such as access to food and water, expansion of endemic diseases as well as an increase of natural disasters and related diseases. The objective of this review is to summarize the current knowledge on climate change effects on military occupational health, military healthcare in a deployed setting, and defense medical logistics.

**Methods:**

Online databases and registers were searched on August 22^nd^, 2022 and 348 papers retrieved, published between 2000 and 2022, from which we selected 8 publications that described climate effects on military health. Papers were clustered according to a modified theoretical framework for climate change effects on health, and relevant items from each paper were summarized.

**Results:**

During the last decades a growing body of climate change related publications was identified, which report that climate change has a significant impact on human physiology, mental health, water- and vector borne infectious diseases, as well as air pollution. However, regarding the specific climate effects on military health the level of evidence is low. The effects on defense medical logistics include vulnerabilities in the cold supply chain, in medical devices functioning, in need for air conditioning, and in fresh water supply.

**Conclusions:**

Climate change may transform both the theoretical framework and practical implementations in military medicine and military healthcare systems. There are significant knowledge gaps on climate change effects on the health of military personnel in operations of both combat and non-combat nature, alerting the need for prevention and mitigation of climate-related health issues. Further research within the fields of disaster and military medicine is needed to explore this novel field. As climate effects on humans and the medical supply chain may degrade military capability, significant investments in military medical research and development are needed.

## 1. Introduction

Human energy consumption based on fossil fuels is one of the reasons for extreme and frequent climate events such as heat waves, heavy rainfall, and droughts increases globally (1). Subsequent health threats, as heath related illness, airway and cardiovascular symptoms due to worsened outdoor air quality, vector-borne, water-related and food-related infections and impaired mental health, force individuals to migrate, regions to reinforce disaster preparedness, nations to secure access to fresh water, and international organizations to implement global warming mitigation policies (2, 3). Recently, the North Atlantic Treaty Organization (NATO) has recognized climate change as a threat multiplier that impacts allied security, in terms of rising coastal water, flooding, increases in temperature and windspeed (4).

The raise in global mean sea level has been reported to be 20 cm between 1901 and 2018, with the largest annual increase between 2006 and 2018 (1). Since 2000, flood-related disasters have increased by 134%, and the number and duration of droughts also increased by 29%. In total, around 74% of all natural disasters between 2001 and 2018 were water related (5). For example, legionellosis incidence in the United States of America (USA) has been rising rapidly in the past two decades, and an increase in monthly hospitalizations for Legionnaires' disease at sites that experienced cyclonic storms have been verified (6). Studies suggests that increased road exposure to traffic-generated aerosols due to warmer and more intensive events of rainfall is one reason for the increase in legionellosis incidence in the USA (7).

In the Arctic region, melting glaciers can increase the risk of previously unknown types of bacteria and viruses appearing and spreading via glacial meltwater (8). As more meltwaters accumulate in lakes and affects sediment in the area, it elevates the risk of zoonotic diseases. Climate change is also predicted to change the type of species that are present in various regions, implying that completely new hosts encounter the viruses or bacteria (9).

Because of climate changes, vector-borne diseases are projected to spread geographically and to increase in incidence. For instance, due to an expected increase of aedes species in the USA, a subsequent increase of vector-borne diseases such as dengue, chikungunya, and Zika viruses are likely (10). In Europe, the vulnerability for malaria infections may increase because of rising temperatures (9, 11). These transmissions are difficult to predict, because they depend on so many factors, such as the type of disease and the type and duration of the weather event (12–15).

Regarding worsened air pollution, the World Health Organization (WHO) estimates that more than 4 million premature deaths each year can be attributed to the effects of outdoor air pollution. In 2016, 58% of outdoor air pollution-related premature deaths were due to ischemic heart disease and stroke, while 18% of deaths were due to chronic obstructive pulmonary disease and acute lower respiratory infections respectively, and 6% of deaths were due to lung cancer (5). The two main contributors to ambient air quality standards–ozone (O_3_) and particulate matter (PM)–interact with radiation that eventually can induce changes in precipitation and regional circulation patterns. Consequently, climate change is expected to degrade outdoor air quality in many areas worldwide by changing its meteorology and by triggering specific amplifying responses in atmospheric chemistry and in anthropogenic and natural sources (16). Air pollution episodes are associated with stagnation events and sometimes heat waves, which are going to be more frequent due to climate change (17).

Increasing global average temperature is making heat waves hotter and more frequent. In the USA, heat waves have been the top cause of weather fatalities, on average, over the past 30 years (18). Consequently, the prevalence of heat related illness is expected to increase. Although exertional heat stroke (EHS) most often strikes populations in hot and humid environments, EHS can also occur in cooler environments, especially when duty requires full personal protective equipment.

As climate change threatens military readiness, operations and strategy, many countries have developed defense policies not only on their own military carbon footprint, but also regarding climate effects on operations and readiness (19–22). Climate affects both medical care and military readiness. NATO Secretary General identified increased occupational health and safety risks as direct effects of climate change (4). Thus, climate change will transform both, military medicine (the medical speciality comprising combat casualty care, military occupational health, veteran healthcare and defense medical war science) and defense medical support (the organization within the armed forces responsible for medical care of wounded or sick servicemen).

While multiple actions are implemented and planned to mitigate greenhouse gas emissions, little is known about the transformation of military healthcare to adapt to climate change (23, 24). This review was conducted to systematically map the research done in this area, as well as to identify possible existing gaps in knowledge. The following research question was formulated:

What is known from the literature about climate change effects on

1. military occupational health,
2. military healthcare in a deployed, austere setting, and
3. defense medical logistics?

## 2. Methods

### 2.1. Protocol

This is a scoping review on climate change transforming military medicine and defense medical support and was reported according to the PRISMA Extension for Scoping Reviews items (25).

### 2.2. Eligibility criteria

To be included in the review, papers needed to measure or focus on specific dimensions of military medicine (combat casualty care, military occupational health, veteran healthcare, military healthcare in a deployed setting, and defense medical logistics) in a context of climate change.

Peer-reviewed articles were included if they were: published between the period of 2000–2022, written in English, involved military employees, and described effects of climate change on policy, healthcare systems, or individual employees. Quantitative, qualitative, and mixed-method studies were included to consider different aspects of climate change effects on military medicine and defense medical support. Papers on societal costs of climate change related to military medicine or defense medical support were also included.

Studies were excluded if they did not fit into the conceptual framework of the study, focused on specific syndromes only (i.e., exertional heat syndrome), or security aspects only (i.e., defense strategies to secure access to water supplies).

### 2.3. Information sources

To identify potentially relevant documents, NLM PubMed MEDLINE and SCOPUS bibliographic databases were searched on August 22^nd^, 2022, for publications published between 2000 and June 30^th^, 2022. The electronic database search was supplemented by searching NATO Science and Technology Organization (STO) scientific publications (https://www.sto.nato.int/publications/Pages/default.aspx) and publications in the Cochrane library for entries or publications published between 2000 and June 30^th^, 2022.

### 2.4. Search

The search strategies were drafted by an experienced librarian and further refined through team discussion. The final search results were exported into EndNote, and duplicates were removed.

The search string for MEDLINE was as follows:

*((“military personnel”[MeSH Terms] OR “military personnel”[Title/Abstract] OR “military medicine”[MeSH Terms] OR “military medicine”[Title/Abstract] OR “military installations”[Title/Abstract])*

*AND*

*(“climate change”[MeSH Terms] OR “climate change”[Title/Abstract] OR “greenhouse effect”[MeSH Terms] OR “greenhouse effect”[Title/Abstract] OR “global warming”[MeSH Terms] OR “global warming”[Title/Abstract] OR “extreme weather”[MeSH Terms] OR “extreme weather”[Title/Abstract] OR “air pollution”[MeSH Terms] OR “air pollution”[Title/Abstract] OR “rising temperature”[Title/Abstract] OR “droughts”[MeSH Terms] OR “droughts”[Title/Abstract])) AND*

*(2000/1/1:3000/12/12[pdat])*

The search string for SCOPUS was as follows:

*(TITLE-ABS-KEY (“military personnel” OR “military medicine” OR “military installations”) AND TITLE-ABS-KEY (“climate change” OR “greenhouse effect” OR “global warming” OR “extreme weather” OR “air pollution” OR “rising temperature” OR “droughts”)) AND PUBYEAR* > *1999 AND PUBYEAR* > *1999*

The search string for all NATO STO reports was as follows:

*Climate change medicine health*

The search string for Cochrane Library was as follows:

*Climate change*

### 2.5. Selection of sources of evidence

**Figure 1:**
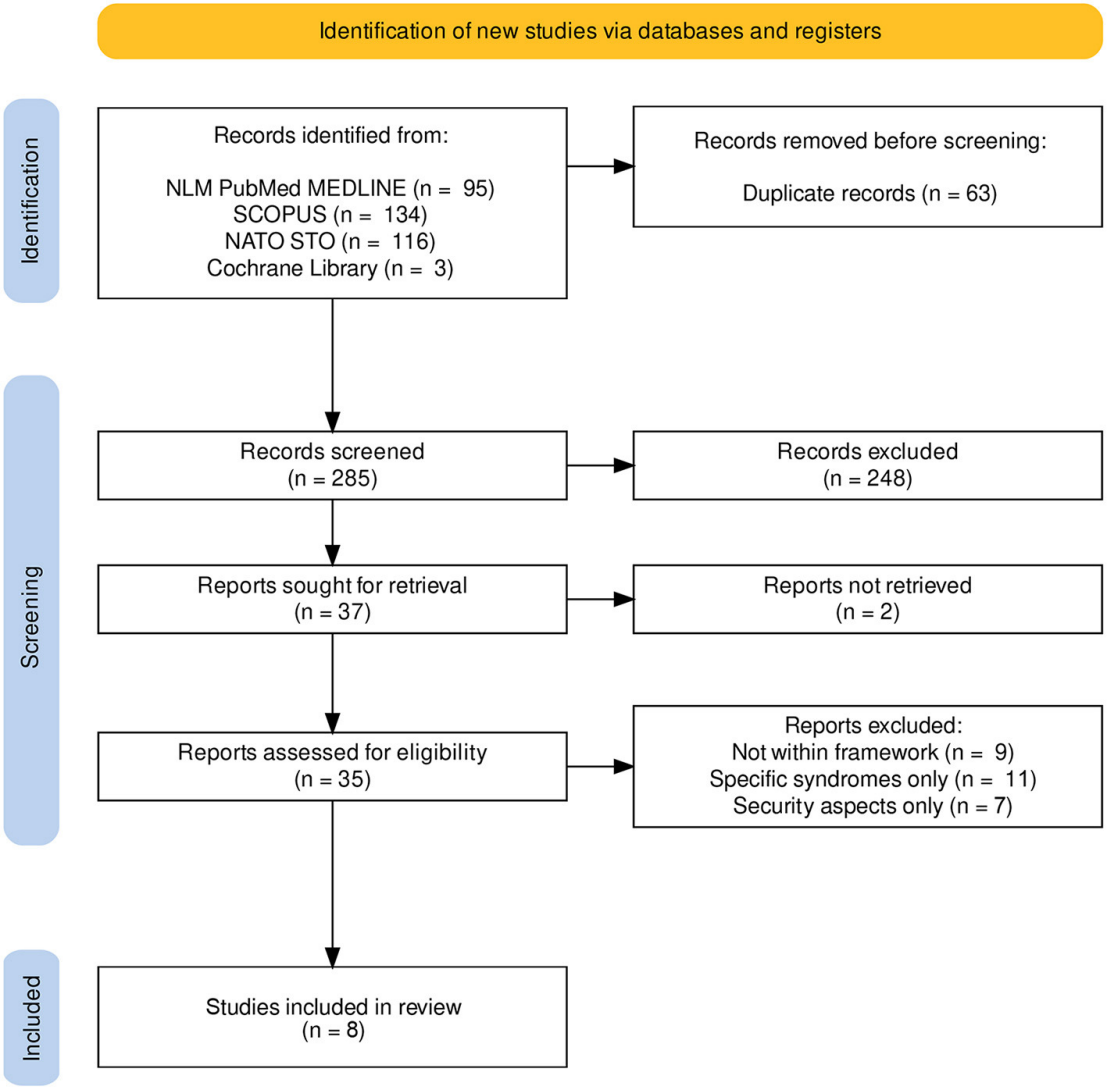
Inclusion flow chart.

Search results were exported into EndNote (version 20.4) and thereafter to Rayyan (https://www.rayyan.ai) to facilitate screening with multiple reviewers (26). Four reviewers sequentially evaluated the titles, abstracts and then full text of all publications identified by our searches for potentially relevant publications. Publications which did not have climate change effects as a primary research question were included if they discussed possible climate change effects on military employees or defense medical support. Disagreements on study selection and data extraction were resolved by consensus, and discussion with other reviewers if needed. The inclusion flow diagram is presented as Figure 1.

### 2.6. Data charting process

Data from included sources were charted using a table recording for each item article, article type, aim, and key messages on military health in climate change.

### 2.7. Data items

Data on article characteristics were summarized in Table 1, engagement characteristics and contextual factors according to the US climate health contextual diagram were summarized in Table 2 (climate drivers, exposure pathways, health outcomes, social and behavioral context, environmental and institutional context, medical support context) (3).

**Table 1:** Key messages of included articles.

**Article**	**Article type**	**Aim**	**Key messages on military health in climate change**
Bozoudis (27) (Greece)	Case study GHP reduction of military hospitals	To create a framework of KPI for the reduction of GHG emissions in the hospital- based military health care.	The highest decrease of GHG emissions is expected to be materialized by the decarbonization of the Greek power sector due to the lignite phase-out and increased share of low carbon fuels and renewable energy sources. Significant emission reduction potential could also be achieved by the replacement of face-to-face hospital visits by telemedicine, primarily by reducing transport-associated emissions. Furthermore, several key performance indicators are proposed as simple and easily monitored metrics of the hospital's performance toward its sustainable low carbon objectives.
Chrétien (10) (USA)	Review heat injuries, vector-borne diseases, and extreme weather that could lead to natural disasters.	Comprehensive approach to planning for military health-related climate change impacts	Exertional heat illness risk among military personnel will increase in a warming climate without additional adaptation measures. For several vector-borne diseases of high military importance, modeling studies suggest increased transmission risk in some areas because of climate change. Since the military is sometimes called on to provide healthcare and public health services when responding to disasters, planning for the increased demand with climate change should include a reassessment of medical relief capabilities and needs.
Connolly (23) (USA)	Narrative review of Global Strategic Trends	This paper considers the potential impact of emerging global strategic trends, including climate change, on in the Future Operating Environment.	The study concludes that future impacts on health service support are neither completely predictable nor predetermined, and there is always a possibility of a strategic shock. Knowledge of vulnerability, however, allows an informed approach to the development and evaluation of adaptive strategies to lessen risks to health.
Erickson (28) (USA)	Survey study on 3657 US Coast Guard responders during the Deep-Water Horizon oil spill disaster.	To document the relationship between environmental heat exposure and heat-related symptoms	Increased prevalence of reported heat-related symptoms among those who experienced higher environmental heat. High-heat personal protective equipment was associated with increased prevalence of reported heat-related symptoms.
Knapik (29) (USA)	Narrative review on heat stroke	To present the pathophysiology, epidemiology, diagnosis, treatment, and prevention of exertional heat stroke (EHS).	EHS most often strikes younger, healthy, and highly motivated individuals who overexert themselves in hot-humid environments. However, EHS can also occur even in cooler environments if the individual is performing intense exercise and accumulates excessive body heat. Key to prevention of EHS and other heat-related injuries is ensuring heat acclimation, adequate work/rest cycles, and proper hydration during activity. Also, certain dietary supplements may have effects on energy expenditure, gastrointestinal function, and thermoregulation that should be considered.
Parsons (30) (Great Britain)	Narrative review on heat adaptation in military personnel	To characterize the physical challenges that military training and deployed operations present, considers how heat adaptation has been used to augment military performance under thermal stress and identifies potential solutions to optimize the risk-performance paradigm, including those with broader relevance to other populations exposed to heat stress.	There is an increased need for monitoring the least fit soldier, who will have the most difficult time (to acclimatize). Furthermore, the most motivated soldiers, who may overdo their physical activity are susceptible to becoming heat casualties. Physical fitness improves physiological responses to exercise in the heat, heat-related illnesses are reported at lower rates in trained personnel than (putatively less well-conditioned) recruits undergoing induction to the Armed Forces, aerobically well-trained participants may be “primed” to heat adaptation with an indirect relationship existing between VO_2_ max and the number of days required to acclimatize.
Petersen (31) (Denmark)	Narrative review on Plasmodium vivax malaria incidence	To discuss the malaria situation in selected countries in the decade before transmission ceased, and discuss the factors which influenced transmission.	Extreme weather events such as hurricanes may lead to migration of large human populations. Evidence that global warning may lead to reemergence or geographical spread of malaria and other vector-borne diseases, in Africa or other endemic areas was clearly provided during the last decade.
Xiang (32) (Australia)	Literature review on military workplace heat exposure risk	To review the characteristics of workplace heat exposure in selected high risk occupations and to provide recommendations for exposure reduction, adaptations and future research directions.	Infantry soldiers have the greatest risk of heat illness, whereas soldiers in administration have the lowest risk. Caucasians are more vulnerable to heat illness than African Americans and Hispanic Americans. Soldiers from the northern US are more susceptible than those from the southern. Female soldiers are at greater risk of heat illness than male soldiers.

**Table 2:** Summary of results according to a modification of the US Climate Health framework (3).

	**Climate driver**	**Exposure**	**Military health outcome**	**Impact on military readiness**
Extreme heat	More frequent, severe, prolonged heat events	Elevated temperatures	Heat related illness, impaired pharmaceutical and blood products supply	Heat-related illness will lead to loss of manpower, impaired decision-making, and an impaired medical cold supply chain
Outdoor air quality	Increasing temperatures and changing precipitation patterns	Worsened air quality (ozone, particulate matter, and higher pollen counts)	Acute and chronic respiratory disease	Respiratory effects will reduce soldier performance and may cause long-term effects with disability.
Flooding	Rising sea level and more frequent or intense extreme precipitation, hurricanes, and storm surge events	Contaminated water, debris, and disruptions to essential infrastructure	Drowning injuries, gastrointestinal and other illnesses	Flooding of fresh-water reservoirs restricts the operational degree of freedom, loss of manpower due to drowning and gastrointestinal diseases
Vector-borne diseases	Changes in temperature extremes and seasonal weather patterns	Earlier and geographically expanded tick activity	Rising incidence of Lyme's disease, tick borne encephalitis, tularemia, and malaria	Degraded performance due to malaria prophylaxis, disability and chronic disease vector borne diseases
Water-related infections	Rising sea surface temperature, changes in precipitation and runoff affecting coastal salinity	Recreational water or shellfish contaminated with *Vibrio vulnificus*	*Vibrio vulnificus* induces diarrhea and intestinal illness, wound and bloodstream infections	Degraded soldier performance due to water-borne infectious diseases, greater effort to secure sufficient drinking water quality.
Food-related infection	Increases in temperature, humidity and season length	Increased growth of pathogens, seasonal shifts in incidence of salmonella exposure.	Salmonella infections and gastrointestinal infections outbreaks	The higher rate of gastrointestinal infections degrades soldiers performance, greater scrutiny needed regarding food hygiene and cold supply chain.
Mental health and well-being	Climate change impacts, especially extreme weather	Level of exposure to traumatic events, like disasters	Distress, grief, behavioral health disorders, social impacts, sleeping disorders	Impaired resilience against other stressors.

Moving from left to right along one health impact row, the three middle columns show how climate drivers affect an individual's or a community's exposure to a health threat and the resulting change in military health outcome. The overall climate impact on military readiness is summarized in the final column.

### 2.8. Synthesis of results

**Figure 2:**
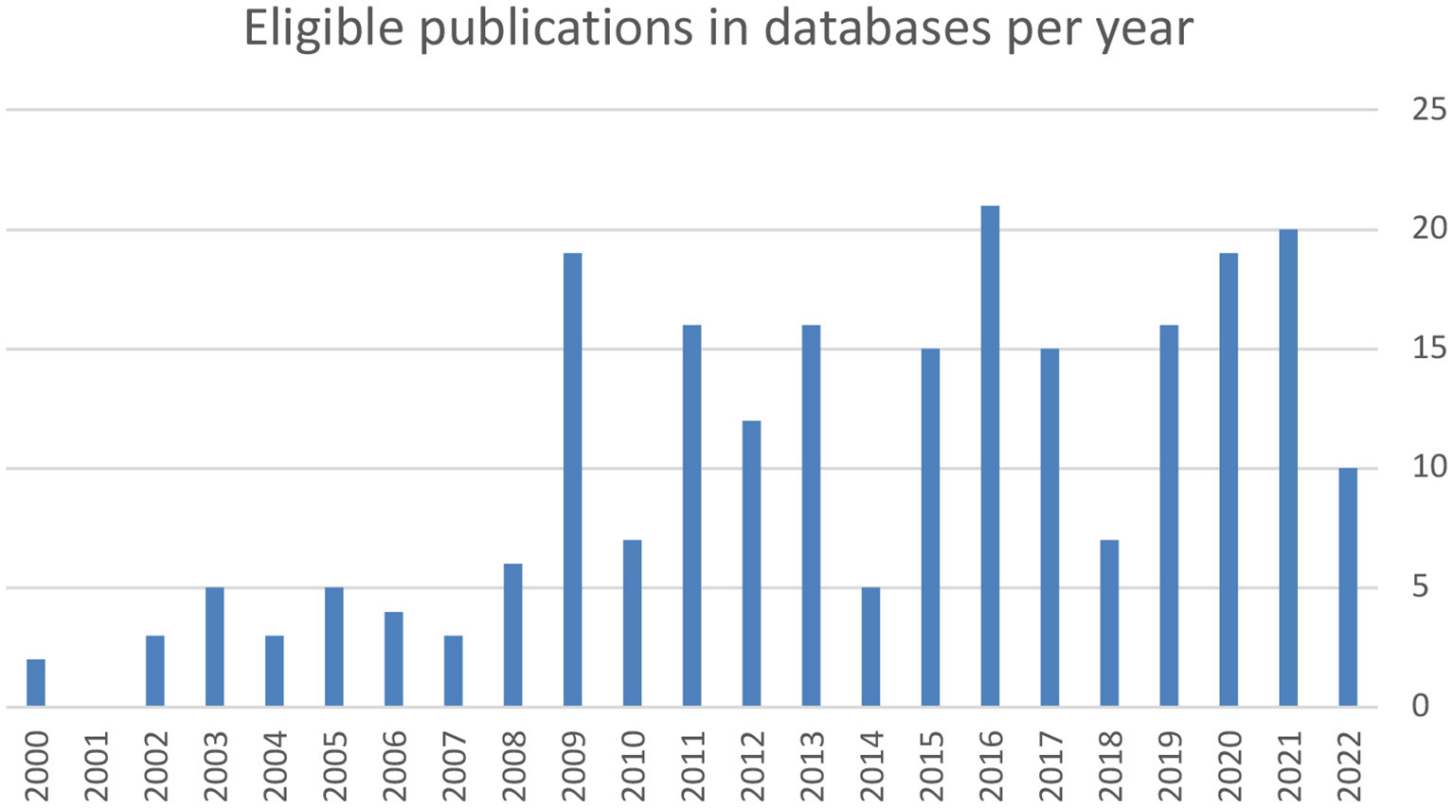
The number of eligible publications found within databases increased linearly between 2000 and 2022 (*r* = 0.73).

The studies were grouped by the types of climate change effects analyzed, and summarized by the type of settings, populations, and study designs for each group, along with the measures used and general findings. Data was presented in a table where climate driver, exposure, military health outcome and impact on in military readiness are described for each climate effect (3). The thematic clustering was performed according to a modified US Climate Health thematic framework in Figure 2 to the themes extreme heat, outdoor air quality, flooding, vector-borne diseases, water-related infections, food-related infection, mental health and well-being and were presented in Table 2 (3).

## 3. Results

### 3.1. Selection of sources of evidence

A total of 229 peer-reviewed articles were screened (95 from NLM PubMed MEDLINE, 134 from SCOPUS), of which after exclusion of 63 duplicates, 166 studies were assessed for eligibility, and topically clustered into 71 occupational hazards and air pollution, 30 military sustainability, 23 climate security policy, 13 tobacco abuse, 12 infectious diseases, 10 historical papers, 9 heat syndromes, 7 water and drought related papers, 3 papers on cold weather operations, and 3 papers on climate related mental health. The annual count of publications on the searched topics increased during the last two decades from about 2 publications annually in 2000 to 20 publications in 2021 (*r* = 0.73, Figure 2). From the NATO STO reports register, 116 publications were retrieved of which 2 fulfilled the inclusion criteria, as they belonged to the research symposium HFM-168 which was on the soldier in cold environment but were excluded as they were not directly related to climate change. From the Cochrane library 3 publications were retrieved, of which none fulfilled the inclusion criteria. Among database and registries search results 8 articles were finally included in the review. The inclusion flow diagram is presented in Figure 1.

### 3.2. Characteristics and results of sources of evidence

Of the 8 included papers 6 were narrative reviews, 1 case study, and 1 survey study. Four papers were from the USA, and from Australia, Denmark, Great Britain and Greece, one publication each was included. The characteristics and results of each included article are summarized in Table 1.

## 4. Discussion

**Figure 3:**
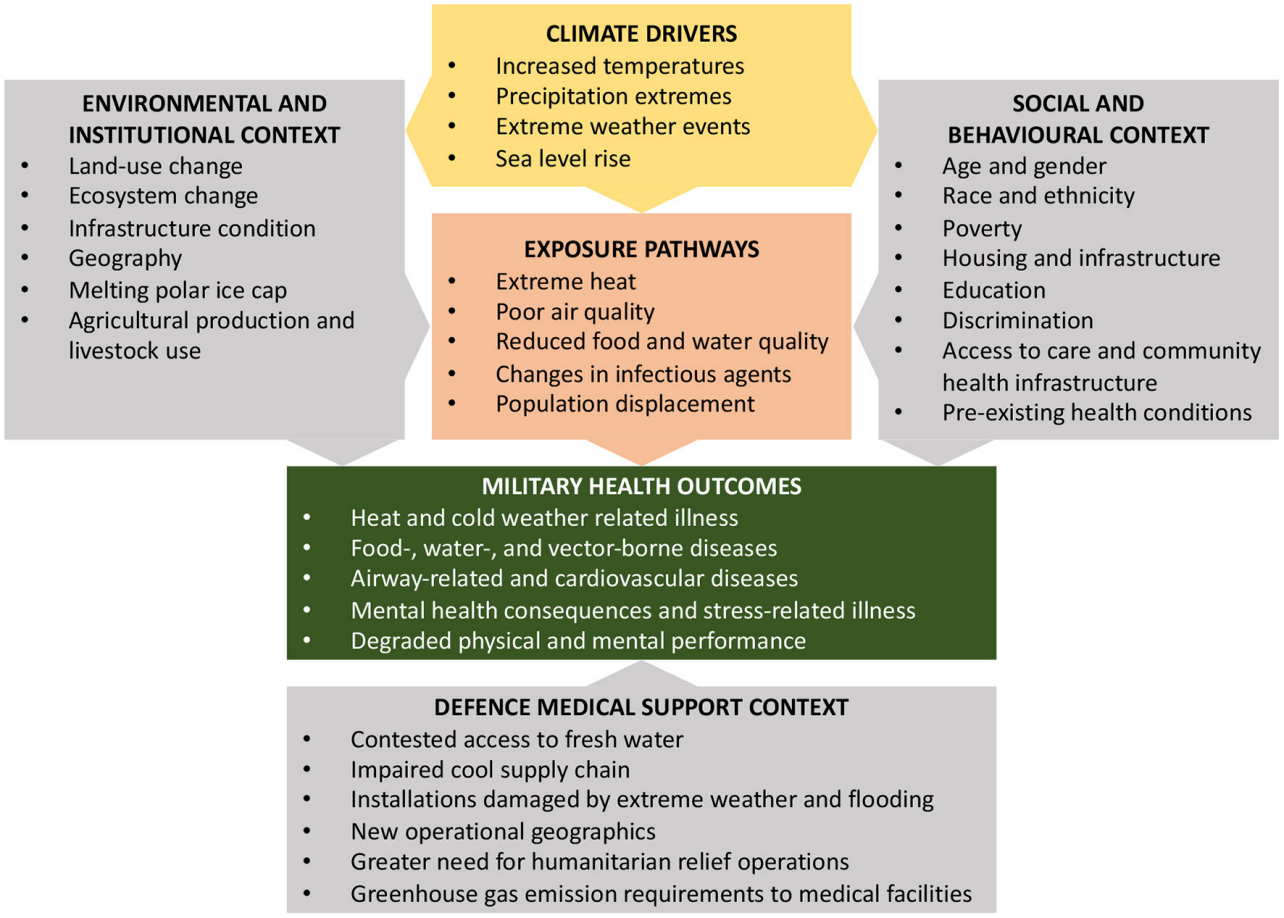
The modified conceptual diagram of the U.S. Global Change Research Program illustrating the exposure pathways by which climate change affects military health outcomes was used as framework for this review (3). According to this framework, exposure pathways exist within the context of other factors that positively or negatively influence military health outcomes, as depicted in the gray boxes.

This is to our knowledge the first review of the available literature on climate change effects on military health and defense medical support. We identified multiple climate change effects in military occupational health, military healthcare in a deployed setting and on military medical logistics and suggested a modified framework for climate change effects on military health in Figure 3. The findings of this study are summarized in Table 2.

### 4.1. Limitations

One of the major biases in defense-related literature search is the restricted access to classified reports. This probably caused a selection bias where some data may not be accessible even though they exist, for example in medical intelligence reports. Still, military medicine has long been treated with lesser confidentiality than technical research areas, and military medical results have a history of publicly accessible scientific reports.

Nevertheless, the authors decided to use the climate change effects framework which was applied by the US government only (3). In comparison to other available frameworks (33), the chosen framework was deemed by the authors to be acceptable for this scoping review, as it simplified the clustering of literature search results and was easily modified to a framework for dedicated military healthcare purposes.

Another bias is the selection of English-language reports, only. This limits searches among reports from large military medical research centers i.e., in Russia or China.

### 4.2. Climate change and military occupational health

#### 4.2.1. Extreme heat

More geographical areas will experience extreme heat causing occupational health hazards, previously only known from operations in tropical areas (10). To allow strenuous exercise during military training, recruits need to get adapted to extreme heat to avoid occupational injury including improved decision-making under heat stress (30, 34). Women seem to be less vulnerable to severe exertional heat illness than men (35). Strenuous exercise in hot environments with a core temperature rise by >1°C does not only affect circulating stress hormones, including catecholamines and cytokines, but also circulating leukocyte counts (36). The health effects of such challenges affect the immune system and may cause several diseases, especially respiratory tract infections.

#### 4.2.2. Outdoor air quality

There is a close relationship between exposure to small particulate matter (PM_10_ and PM_2.5_) and increased mortality or morbidity, both daily and over time. Repeated exposure can lead to increased permeability in lung tissue, elevating susceptibility to viral and bacterial pathogens and aggravation of the severity of chronic lung diseases. Since PM may cause inflammation of lung tissue, there is a high risk of changes in blood chemistry that impact heart function in several ways. Several studies have indicated a correlation between both upper and lower airway symptoms and the military occupational health. For example, it has been shown that exposure to PM_2.5_ is a major independent contributor to the development of chronic sinusitis in the active-duty military population (37). The degradation of ambient air quality due to climate change will likely add to the risk of airway symptoms and perhaps also cardiovascular symptoms among military employees (17).

#### 4.2.3. Flooding

Sea level rises pose an existential threat to nations with large coastal areas–not only due to loss of land surface but also through saltwater intrusion in drinking water wells. Some small island nations are projected to become mainly uninhabitable already during the 21^st^ century.

The risk of flooding of coastal installations and fortifications in areas with high precipitation requires that the design of military installations is adjusted to climate change. Storm surge and coastal erosion related to climate change will add to the challenges of military installations and the health of servicemen. Flooding related risks are, besides the effects on water quality, a greater incidence of drowning accidents, hypothermia, impaired medical evacuation possibilities, and damaged medical facilities.

#### 4.2.4. Vector-borne diseases

Surveillance of vectors and related diseases will become more important for military epidemiologists. The use of repellants and mosquito nets will become mandatory in more areas, requiring education and a new set of skills for military personnel in their home countries (9).

The effect of rising temperatures on the spreading of infectious diseases has been studied more extensively during the last decades. Vector-borne infections are expected to be the most climate-sensitive subset of all infectious diseases and have sensitivity to the greatest number of climate drivers. Arboviruses such as dengue, Zika, yellow fever and chikungunya viruses are transmitted by Aedes mosquito species, which have been spread into new areas due to climate changes, globalization and international trade. These diseases poses serious concern for public health services, not at least the large potential of Aedes mosquitos to transmit viruses in urban settings (38). In a recent study, researchers have also systematically mapped how, not only viruses, bacteria and parasites are affected by climate change, but also how insects and allergens are affected by rising levels of greenhouse gases (39).

In the Nordic countries, zoonoses such as Lyme disease, tularemia and nephropathia epimedica have been increasing for a long time due to habitat destruction and reduced biodiversity, and the increase is expected to accelerate further due to prolonged and more frequent mild winters (40). Since military personnel spend a relatively large part of their working time in field environment, the increased risk of infection poses a threat to the health of military personnel.

#### 4.2.5. Water-related infections

Water-borne pathogens often act through two major exposure pathways: drinking water and recreational water use. Higher mean temperatures trigger more intense precipitation events. These events can result in run-off and loading of coastal waters with pathogens, that may adversely affect the health of people using recreational water. Since the risk of gastroenteritis and respiratory infections due to recreational water use are much higher during the rainy season than during the dry season, more intense precipitation during rainy seasons will likely increase that risk, both for the general population and for military personnel (12).

Depending on water treatment capability, the quality of drinking water and hygiene water can be affected due to climate change as well. Precipitation extremes have been linked to both outbreaks and sporadic cases of waterborne illness. For example, studies have shown a link between heavy rain and turbidity to population-level risk of sporadic cryptosporidiosis and giardiasis (41). In developing countries, events such as flooding have been associated with epidemics of cholera and other diarrheal diseases. In Europe, flooding has rarely been associated with an increased risk of water-borne disease outbreaks, although a few exceptions exist (14). Thus, the extent to which climate change will increase the risk for infections from drinking water among military personnel depends on several factors, such as geography, treatment capability and socioeconomic status.

#### 4.2.6. Food-related infection

Climate change directly affects food safety, as rising temperatures are associated with a high number of pathogens and bacterial toxins (42). Researchers found in several European countries a linear association between temperature and the number of reported cases of salmonellosis above a threshold of 6°C (43). Vibrio cholerae is endemic in only certain regions of the world, even though it is commonly found all over the world. A complex interaction of abiotic factors, including temperature, salinity, iron concentration, and sunlight, influences V. cholera toxin production (44).

Furthermore, climate change will change the amount and type of mycotoxins in our food. In warmer climate the presence of fungi and mycotoxins such as A. flavus and aflatoxin will increase (45).

Climate change effects on food safety will change both the need for cool storage and the preparedness for food-related infections. As food poisoning has a dramatic impact on soldier performance prevention and monitoring of upcoming gastrointestinal infections will become an important task for defense medical support.

#### 4.2.7. Mental health and well-being

There are few, if any papers specifically investigating military mental health due to climate change over extended time periods, and assumptions in review papers are not entirely based on scientific evidence. Thus, the ability to draw scientifically conclusions with respect to climate change related mental health or mental diseases in military settings are very limited. Symptoms related to mental health in an occupational military setting may be related to both regional climate changes, as well as mission and training. In general, the mental health may not be as jeopardized as in civilian population, due to selection bias of personalities and mental capacities, and the systematic training of military personnel expected to operate in challenging conditions. However, a previous qualitative study among civilians during an annual half-marathon race found a perceived risk profile among runners who collapsed and required hospital care (46). Their personality self-report included descriptions such as being stubborn, ambitious, disciplined and performance oriented. Such personality traits are without a doubt not rare within the military staff, and may relate to profession specific physical and mental outcomes of the military staff performance due to temperature change. Also, military personnel already operate in extreme weather conditions, adding a certain strain on their mental health, that civilians normally are not exposed to.

It is shown that with an increasing air temperature above an optimum, physical and cognitive performance decreases. Cognitive performance may decrease along with increased temperatures (47), and especially complex cognitive tasks seem to be most vulnerable to physical heat in soldiers (48). Also, climate related supply chain disruptions may occur putting basic needs of food and clean water, at risk, both during deployment and in regular training, elevating stress and general anxiety, thus interfering with several aspects of cognitive performance even if military resilience training has been performed (49).

### 4.3. Climate change and military healthcare in a deployed, austere setting

#### 4.3.1. Extreme heat

Due to climate change, scenarios in which unacclimatized personnel need to either serve at short notice in areas with severe climate or to over-ride intrinsic or extrinsic thermal safeguards in the face of physical or tactical threats will become much more frequent. These events will cause more military personnel with heat stroke or other heat related symptoms and ultimately degraded capability (30).

The NATO STO Research Task Group on Management of Heat and Cold Stress Guidance to NATO Medical Personnel identified multiple heat acclimatization strategies prior to deployment (50).

#### 4.3.2. Outdoor air quality

Partly because of deployment in areas with polluted air, military troops worldwide have a long and extensive history of airway related diseases and symptoms (36, 37, 51). The distribution of PM air pollutant typically encountered by deployed military personnel, such as desert dusts, burn pit smoke, oil well fires and local industrial pollutants, are expected to be affected by climate change, in a way that may increase the risk for lung disease, no matter whether the air pollution is caused by the military itself or not.

It is therefore important that contingency hospital care is aware of and have a redundance for an increased risk for airway related diseases and symptoms among deployed personnel. As location, duration, and frequency of deployment contribute to the risk of respiratory symptoms they should be part of every soldier's medical history (36).

#### 4.3.3. Flooding

The greater risk for flooding of lowlands through climate related precipitation and storm surge requires an increased awareness of climate related aspects when planning future operations. The scenario of operating bases being flooded, decreasing military capability, is a likely scenario in future operations in deployment. As flooding causes sanitary challenges with a high risk of drinking water contamination, the importance of military medical presence during operational planning is obvious. Therefore, students attending medical-programs or health-programs need to be trained in preventive medicine aspects related to natural disasters and in austere or deployed environment.

#### 4.3.4. Vector-borne diseases

Deployed soldiers are exposed to vector-borne diseases endemic in the deployment area. During World War I and II, soldiers in South East Asia and the Mediterranean have been infected with plasmodium species and brought the malaria to their home countries (52, 53). Meticulous preventive medicine measures–including pesticides and chemoprophylaxis–were successfully containing and in some countries eradicating the disease. But, due to climate change, malaria has resurfaced both due to migration of infected populations, as well as due to increased vector populations in warmer climate (31).

Ingestion of pesticides, as permethrin and pyridostigmine bromide, may affect the human microbiome and may cause chronic inflammatory diseases (54). It is likely that the greater use of pesticides will lead to a higher incidence of adverse effects, which need to be followed cautiously by the medical professions.

#### 4.3.5. Water-related infections

Effective water treatment plants in deployed settings (i.e., comprising reverse osmosis, ultrafiltration, or microfiltration technique) normally provides drinking water of high quality to deployed personnel. However, unpredictable rainfall, increased flooding and extreme precipitation in deployed areas will inevitably increase the risk of water-borne diseases and creates conducive breeding grounds for insect vectors at or close to the deployment setting. Moreover, in case of drought, limited and deficient water supplies can perpetuate unhygienic conditions, that might increase the risk of several water related diseases, for example diarrheal disease. Thus, for each contingency hospital care, an awareness of and redundance for increased risk for water-related infections is important.

#### 4.3.6. Food-related infection

Warmer climate impairs food safety (55). For instance, the microbial quality of raw milk is threatened by warmer climate, as raw milk contamination with E. coli is dependent on temperature (56). Even the presence of micro-organisms that produce food-borne illnesses such as salmonella and campylobacter may increase with rising temperatures (55). Especially in deployment a well-controlled and functional cold chain for food supply is vital for preventing food-borne illness (57).

As the climate change increases the rate of worm parasites in wild and domestic animals, the quality of the available meat is highly dependent on the ability to perform proper diagnostics for parasites (58). Military personnel in deployment who are dependent on locally acquired or hunted meat need high medical intelligence capabilities as well as parasite detection capabilities.

#### 4.3.7. Mental health and well-being

Future challenges in military mental health are related to both environmental changes involving climate related changes in heat, humidity, cold, malnutrition and natural disasters or extreme weather events. These factors ought to be part of military medical intelligence preparations. These conditions can all affect the mental state via mechanisms of sleep disturbances (lack of sleep or disturbance of circadian rhythms, contributing to anxiety related behavior). Interestingly, in some military settings report of pain is stigmatized, thus complaints about sleep problems, is more accepted as a medical issue (59). In war, lack food and water intake may be part of the logistic disruptions, thus elevating levels of stress and anxiety (starvation is one of the oldest weapons of war being deployed or in war). Calorie deprivation and forced evasion is a unique and severe stress. Performance decrements and psychological changes are evident within 24 h of cessation of nutritional intake. Overall, in any form of environmental stressor on the central nervous system of the operating military personnel, a failure to regulate prolonged periods of excessive cortisol levels may not only elevating risk of mental problems related to both anxiety and depression, but also reduce cognitive capacity, possibly by hippocampal sensitivity (60), being the gateway to complex cognitive functions (61).

### 4.4. Climate change and defense medical logistics

#### 4.4.1. Access to fresh water

Poor water quality and sanitation constitute perhaps the most critical threats to health and safe healthcare (62). Poor water quality does not only affect the availability and quality of freshwater, but also affects the future demand for fresh water. Between now and 2,071, up to 14 % of the global population is estimated to experience a severe reduction in water resources given, a global warming up to 2.7°C (63). Water availability for countries in Europe will likely be affected by increasing runoff and flood magnitudes (64), although perhaps not pronounced at higher latitudes, e.g., in most of Finland, northwestern Russia and northern Sweden (65).

In addition, a global warming of 2°C will also impact water quality in lakes, watersheds, and regions, in terms of a possible increase in chloride levels, that can result in chloride concentrations well above drinking water standards in some regions (66). In a similar fashion, expected increase in leakage of nitrogen and phosphorus from agricultural areas to nearby lakes might affect the usability of fresh water (67). In addition, recent studies on local or regional areas already indicate a decrease in dissolved oxygen in some freshwater basins (68).

Access to fresh water has a profound relevance for military operations in terms of hygiene, healthcare and defense medical logistics. Further research is needed to define defense medical supply system requirements for safe fresh water in times of climate change.

#### 4.4.2. Temperature-sensitive medical supplies and devices

The medical treatment chain requires full access to medical supplies: pharmaceuticals, infusions, diagnostics, blood products, as well as medical devices for prehospital care, outpatient clinic, surgical theater, intensive care unit and ward. Many of these medical supplies are temperature sensitive and lose their effect and may cause harm if the temperature chain is interrupted (69, 70). Climate change forces military pharmacists to improve their fridge and freezing capacity in the cold supply chain, as well as having a higher storage turnover.

Blood products as erythrocyte concentrates and thrombocytes are highly temperature sensitive. As mass casualty incident preparedness includes storage of blood group 0 erythrocytes, plasma and thrombocytes, the storage and transport of these products in warmer areas has to rely on highly effective cooling systems (71).

Many medical devices depend on semi-conductor technology that needs to be cooled during operation. Climate change will be a driver of new semiconductor cooling technology development, and medical devices cooling needs to develop accordingly.

#### 4.4.3. Increased need for natural disaster relief

In many countries the armed forces will respond to natural disaster relief in support of the civilian disaster response (10). As the number of natural disasters will continue to increase due to climate change, disaster relief operations will consume more medical and logistic support resources. Climate change acts as a risk multiplier for armed conflict (72). Due to climate related conflicts, disaster relief operations will to a greater extent take place in conflict-ridden countries, putting both military and civilian disaster medical relief personnel at risk for both physical and mental problems, raising the need for regional psychosocial and mental health programs (73). Moreover, due to security issues, a greater deployment of armed forces with medical support to humanitarian relief operations may be needed as well (74–76). Longer-term planning of military capabilities needs to account for humanitarian support during climate change related peace-keeping operations.

#### 4.4.4. New operational geography

The melting ice in the arctic region opens for new waterways allowing shorter sea transport routes between Asia and Europe. This will increase the need to protect these waterways, requiring military presence in the Arctic North more than ever before (4). The changes in operational geography will affect military medicine as well: Prehospital and emergency care will be performed in arctic climate, with consequences of a higher risk of hypothermia, increased bleeding risk, and malfunctioning medication and medical devices. Defense medical support thus needs to establish a medical supply chain fit for Arctic operations. Occupational medicine will need to focus on preventive measures to protect operators in cold environment by leadership development, training, acclimatization, and equipment (77).

#### 4.4.5. Greenhouse gas emissions of military healthcare facilities

Medical facilities use large amounts of electric energy, produce large amounts of waste, even potentially hazardous waste, and leaves a significant contribution to greenhouse gas emissions (78). Indeed, the healthcare sector is responsible for about 4.5% of greenhouse gas emissions worldwide (79). Therefore, it is reasonable to investigate the carbon footprint of military healthcare installations. Bozoudis *et al*. found hospital electricity to be with 63% the largest contributor of greenhouse gas emissions in a Greek military general hospital, followed by fossil fuels with 32%, refrigerators with 5% and air conditioning with 4%. Waste disposal accounted for < 1% of the carbon footprint of the military hospital. By using a more environmental mix for the electric energy consumption, switching to more fuel-efficient heating systems, and the addition of solar panels on hospital roofs and parking lots, the carbon footprint of military hospitals can be reduced.

Additionally, alternative ways to meet and diagnose patients are available, for instance by telemedicine, diagnostic drones or drone based medical delivery, which may reduce the carbon footprint of healthcare (80, 81). Further research is needed to identify further possibilities for reduction of the military healthcare carbon footprint.

## 5. Conclusions

This scoping review identified multiple knowledge gaps regarding the impact of climate change on military medicine and defense medical support. There is a body of literature which supports the assumption that military medicine needs to adapt to extreme temperatures, worsened outdoor air quality, flooding of installations, higher rates of vector-borne diseases, water-related and food-related infection, as well as impaired mental health caused by climate change. Defense medical logistics need to adapt to climate change, when fresh water supply is contested, the cold supply chain for pharmaceutical and blood products is impaired, when military support to natural disaster relief is requested, new operational areas emerge, and military healthcare facilities must reduce their carbon footprint.

If military medicine and defense medical support fail meeting future energy-saving demands and cope with environmental challenges, the military capability and the performance of armed forces personnel may decline along with increasing climate change effects. In other words, the armies with the greatest versatility in adaptation to climate change will have an advantage on the battlefield, which likely will be measurable in tactical performance and higher troop morale.

As next steps defense research and development efforts must be directed toward a better knowledge of climate change impact on military health and defense medical support. According to our understanding, the earnings from investments in military medicine transformation toward improved climate change adaptation will be stronger resilience and tactical superiority.

## Data availability statement

The original contributions presented in the study are included in the article/supplementary material, further inquiries can be directed to the corresponding author.

## Author contributions

YR designed the study and collected the data. YR, AK-M, NA, CS, and FT performed the analysis and collaboratively wrote the paper. All authors contributed to the article and approved the submitted version.
